# Impact of the Drug Prices Control Order (2013) on the Utilization of Anticancer Medicines in India: An Interrupted Time-Series Analysis

**DOI:** 10.7759/cureus.26367

**Published:** 2022-06-27

**Authors:** Bhavna Sharma, Aashna Mehta, Habib H Farooqui, Himanshu Negandhi, Sakthivel Selvaraj

**Affiliations:** 1 Health Economics and Outcomes Research, Skyward Analytics, Gurugram, IND; 2 Health Economics, Financing, and Policy, Public Health Foundation of India, Gurugram, IND; 3 Population Medicine, Qatar University College of Medicine, Doha, QAT; 4 Public Health Research, Public Health Foundation of India, Gurugram, IND

**Keywords:** interrupted time series, drug pricing policy, anticancer, drug prices control order, dpco

## Abstract

Objectives

The National Pharmaceutical Pricing Authority introduced a series of Drug Prices Control Orders since 1970 to regulate the prices of essential medicines in India. This study evaluated the impact of the Drug Prices Control Order of 2013 on the utilization of anticancer medicines in the Indian private sector.

Methods

We used monthly sales audit data for a period of 2012-15, provided by Intercontinental Medical Statistics (IMS) Health. Through interrupted time series design and segmented regression models, we estimated the change in utilization of anticancer medicines following the drug pricing policy implementation.

Results

Of 1556 anticancer drug packs, 22.3% (n= 347) were price-controlled. The policy led to an immediate monthly reduction of 27.3% (95% CI -38.6%, -13.9%; p=0.001) and a long-term monthly reduction of 0.7% (95% CI -1.6%, 0.3%; p=0.16) in price-controlled formulation’s utilization. In the final study month, the price-controlled formulation’s utilization was 5.03 thousand standard units lower than what would have been expected without the policy. Melphalan showed the highest immediate reduction, and alpha-interferon showed the highest long-term reduction in utilization.

Conclusion

Drug prices control order 2013 caused an immediate and long-term decline in the utilization of anticancer medicines in the Indian private sector. However, study data was limited to a specific part of the Indian anticancer drug market, which must be considered when interpreting findings.

## Introduction

Globally, cancer-related economic impact has been estimated to range between US$ 290 billion to US$ 900 billion [1]. Households in India bear high out-of-pocket expenses for cancer treatment and care on account of low governmental allocation for the public health sector and a poor health financing system [2]. Of the total expenditure of cancer treatment, medical expenditure constitutes around 80-90% [3]. A major contributor to the high medical expenditure is the rising prices of anticancer drugs. Anticancer drug prices have increased by almost 10 times in the last decade and account for about one-fourth of total cancer costs [4]. Moreover, the low availability of medicines in the Indian public sector compels the patients to obtain medicines from the private sector at a much higher price [5,6]. The problem is further exacerbated as 90% of the Indian population purchases medicine through out-of-pocket payments [5,6]. A global comparison of anticancer drug prices conducted in 2016 showed that although the prices of anticancer drugs are highest in the U.S, their affordability is the lowest in India by a huge margin [7]. An interplay of high out-of-pocket expenditure and a lack of insurance coverage forces many households to resort to distressed financing through borrowings, contributions from friends or relatives, and even the sale of household assets [1,8]. The “financial toxicity” associated with cancer treatment not just renders cancer care inequitable and unaffordable but also deters patients’ clinical condition, so much so that bankruptcy due to cancer treatment has been recognized as a risk factor for the early death of cancer patients [9,10].

The soaring price of medicines has been receiving growing attention from regulatory authorities for decades. The primary regulatory body concerned with the price regulation of medicines in India is the National Pharmaceutical Pricing Authority (NPPA) [11]. Although the NPPA was established under the Ministry of Chemicals and Fertilizers in 1997, the prices of medicines in India have been regulated since the 1970s through a series of Drug Prices Control Orders (DPCOs) [11,12]. The DPCO is a government-issued order promulgated under Section 3 of the Essential Commodities Act of 1955. The first DPCO was introduced in 1970, which was subsequently revised in 1979, 1987, 1995, and the most recent revision in 2013 [12,13]. DPCO 2013 was enforced on the 13th of May, 2013, to implement the National Pharmaceutical Pricing Policy (NPPP) of 2012 [14]. The objective of the policy was to harmonize pharmaceutical innovation and improve the accessibility of medicines for the general population [14]. DPCO 2013 aimed to do so through its three key principles, which contrast it with its previous counterparts [14]. One, DPCO 2013 is formulation-specific and not drug-specific, i.e., it regulates the prices of certain strengths and dosage forms (such as tablets, capsules, etc.) of a bulk drug in contrast to the immediate prior order (DPCO 1995), which regulated prices of the bulk drug. A bulk drug is an active drug substance, which alone or in combination with inactive ingredients form the final medicinal product (known as formulation). Two, DPCO 2013 replaced the cost-based pricing of the earlier orders with market-based pricing (MBP) approach and computed a ceiling price of each formulation. Ceiling price refers to the maximum price of a formulation at which it is sold to the consumer, excluding the local taxes. Ceiling price calculation using MBP involved calculating an average price to retailer (PTR) of medicines having a market share of more than equal to 1%. A fixed 16% margin to the retailer was then added to the average PTR to obtain the ceiling price of a formulation. Also, unlike the previous versions of DPCO, which lacked provision for revising the prices, the current DPCO revises the ceiling price of each formulation on the 1st of April of every year, based on the wholesale price index (WPI) of the formulation for the preceding year. Three, the DPCO 2013 regulates the prices of essential formulations only, i.e., the formulations listed in the National List of Essential Medicines (NLEM) [14]. Thus, when DPCO 2013 was enforced, it regulated the prices of 348 essential medicines listed in NLEM 2011, of which 40 were anticancer drugs (corresponding to 63 anticancer formulations). But currently, the policy regulates the prices of 376 essential medicines listed in NLEM 2015, of which 59 are anticancer medicines (corresponding to 108 anticancer formulations) [15,16].

The ceiling prices of essential anticancer formulations were notified gradually, rather than at a single time point. NPPA notified ceiling prices of anticancer formulations over a period of 14 months (June 2013 to July 2014), which denotes the implementation period of the policy (DPCO 2013) for anticancer formulations. But, as per the norms of the policy, each ceiling price notification was followed by providing an additional 45 days to its manufacturer to fix/revise the maximum retail price (MRP) of the formulation. Therefore, for anticancer formulations, the price adjustment period was extended from August 2014 to the end of September 2014 [14].

Policy evaluation studies for anticancer drugs have been conducted in countries of varying income groups, including the U.S., Italy, and China [17-20]. But, most of these studies evaluated the impact of the addition of anticancer medicines in insurance coverage or modification of existing cancer-related reimbursement schemes. These studies demonstrated either no systematic change or an improvement in the utilization of anticancer medicines after the implementation of the policy [17-20]. In 2017, an Indian study evaluated the impact of Drug Prices Control Order 2013 on the average price of an antidiabetic formulation (metformin 500mg). The study found that, as compared to unregulated metformin, the price of regulated metformin formulations increased prior to the policy implementation, which later declined after the policy was implemented [12]. Another Indian study in 2019 evaluated the impact of DPCO 2013 on statins and found a shift in utilization from unregulated to regulated statins after the policy was implemented [21]. Thus, to the best of our knowledge, this is the first attempt at studying the impact of DPCO 2013 on the utilization of anticancer medicines.

In our study, we aimed to evaluate the impact of drug pricing policy (DPCO 2013) on the utilization of anticancer medicines in India. Our study objective was to quantify the impact of DPCO 2013 on the utilization of anticancer medicines in the private sector of India. We hypothesized that as compared to the pre-policy period, the utilization of anticancer medicines in the retail medicine sector of India would have increased after the drug pricing policy (DPCO 2013) was implemented.

## Materials and methods

Data source

Our study used sales audit data by Intercontinental Medical Statistics (IMS) Health, which is currently known as IQVIA. It is a for-profit organization that collects and provides data on pharmaceutical market intelligence in over 100 countries around the world. The data consisted of monthly sales (values and volumes) of pharmaceutical products, collected from a panel of 5600 stockists for a period of five years (2012-16). The data was collected across different regions of India, which was then extrapolated to provide estimates of overall national sales in the private sector. The sales data included sales made by the stockists to hospitals, retailers, and dispensing doctors. The monthly sales data in the dataset was recorded at the level of individual drug packs, and the drugs were classified into specific therapeutic levels, i.e., supergroup, group, and subgroup, using the European Pharmaceutical Market Research Association (EphMRA) classification system.

Outcomes

The dependent variable in our study was cancer medicine’s utilization. We assumed monthly sales volume to be a proxy measure of utilization in our study which was expressed in standard units (SUs). One SU refers to the smallest dose of a formulation, such as one tablet or capsule for oral solids, one vial or ampoule for injectables, and so on.

Study design

We used an interrupted time series (ITS) design in our study. ITS is considered the strongest quasi-experimental design as it balances the trade-offs of observational designs (which possess limited ability to establish causation) and randomized controlled trials (which are resource-intensive and often impractical) [22]. ITS is particularly useful for studying “natural experiments” in real-world settings. In our study, the natural experiment under investigation was the Drug Prices Control Order of 2013 [22-24].

Statistical analysis

Although our data source spanned across five years (2012-2016), we excluded 2016 observations as NLEM was revised in 2015, which could have changed the market dynamics in 2016. Hence, to obtain estimates unaffected by the 2015 NLEM revision, we confined our study period to January 2012 to December 2015. The implementation period of DPCO 2013 divided our total study period (Jan-12 to Dec-15) into three segments: pre-intervention period comprising of 17 monthly data points (Jan-12 till the end of May-13), intervention implementation period consisting of 14 monthly data points (Jun-13 till the end of Jul-14), and post-intervention period formed by 17 monthly data points (Aug-14 till the end of Dec-15).

We descriptively assessed the yearly trend of sales volume and value using summary measures (mean ± SD or median ± IQR), the annual growth rate of cumulative sales (in %), and the compound annual growth rate of cumulative sales (in %). We also assessed monthly sales trends graphically by plotting them against time.

In order to quantify the impact of DPCO 2013 on the utilization of anticancer medicines, we used segmented linear regression analysis and approximated a “line of best fit” separately for data points before and after the intervention was implemented.

Based on a previous study assessing the impact of drug pricing policy on statins in India, we hypothesized a priori that DPCO 2013 can create an impact by altering both the level and trend of utilization of anticancer medicines. Thus, in order to obtain post-intervention estimates which are unaffected by pre-existing secular trends, we created three independent variables, i.e., time, intervention, and time after the intervention. Time was a continuous variable, which represented time from the beginning of our observation period. Thus, it ranged from 1 to 48, depicting each month of our total study period of four years (2012-2015). The intervention was a binary variable, coded zero for the pre-intervention period and one for the post-intervention period. Time after intervention signified time elapsed after the intervention. Thus, time after intervention was coded zero for the pre-intervention period, and it was a continuous variable from the beginning of the post-intervention period.

The regression equation was as follows:

Y_t_ = β_0_ + β​​​​​​_1_Time + β_2_Intervention + β_3_Time after Intervention + ε_t_

where,

Y_t_ : Dependent variable, i.e., logarithm of sales volume of anticancer medicines;

β_o_ : Baseline level (i.e., y-intercept) of the dependent variable at time zero (i.e. Jan 2012)

β_1_ : Baseline trend (i.e., slope) or growth rate of the dependent variable at time t, independent of the intervention effect;

β_2_ : Immediate change in the level of the dependent variable after intervention;

β_3_ : Long-term change in the trend of the dependent variable after intervention;

ε_t_ : Error term at time t, which denotes the random variability unexplained by the model.

As post-estimation tests, we checked our regression models for autocorrelation visually using residual vs. time plot, autocorrelation function plot, and partial autocorrelation function plot. We further used statistical tests for detecting autocorrelation, namely the Durbin-Watson statistic and the Breusch-Godfrey test. We did not test for seasonality as cancer is a chronic condition, and treatment is usually required for a lifetime.

Our primary analysis tested the impact of DPCO 2013 on the utilization of price-controlled anticancer formulations only (Model 1). Since the 45-day adjustment period marked a gradual introduction of the pricing policy in the market through revision of MRPs, it had the potential to confound our results. Thus, to assess if the adjustment period altered the policy impact on the price-controlled market, we conducted the sensitivity analysis by running an alternate model after excluding the adjustment period sales volume of price-controlled formulations (Model 2).

In addition to altering the implementation period, through our sensitivity analysis, we also assessed the impact of the policy on anticancer formulations which did not come under the purview of DPCO 2013, i.e., price not controlled anticancer formulations (Model 3). We further repeated the model after excluding adjustment period utilization (Model 4).

After building actual models, we introduced a counterfactual model, which predicted the outcome in a scenario wherein the pricing policy had not been implemented. This model assumed that in the pre-policy period, a linear relationship existed between utilization and time, which would have continued unaltered in a linear fashion in the absence of policy.

Finally, we calculated the absolute policy effect, which referred to the difference in outcome brought by the intervention (actual model) as compared to what would have continued without the intervention (counterfactual model). The absolute policy effect was assessed visually by plotting the predicted outcomes of actual and counterfactual models in a single graph. Mathematically, the absolute policy effect was calculated by subtracting the predicted utilization of the counterfactual model from the predicted utilization of an actual model.

Additionally, we also assessed the impact of the policy (DPCO 2013) on individual price-controlled anticancer formulation through subgroup analysis. The implementation period used in models for subgroup analysis varied for each anticancer formulation and included the 45-day price adjustment period.

We performed statistical analysis using Stata version 15 (StataCorp LLC, College Station, Texas) software.

Patient and public involvement

Patients or the public were not involved in this study, which is based on secondary data.

## Results

There were 1556 anticancer drug packs in our study data, of which 22.3% (n=347) drug packs were price-controlled, while 77.7% (n=1209) drug packs did not fall under the purview of pricing policy (DPCO 2013). Of 40 essential anticancer drugs (i.e., 63 anticancer formulations) listed in NLEM 2011, price-controlled drug packs in our data (n=347) corresponded to 45 essential anticancer formulations (list of included and excluded formulations, and their ceiling price notification period presented in appendix).

**Figure 1:**
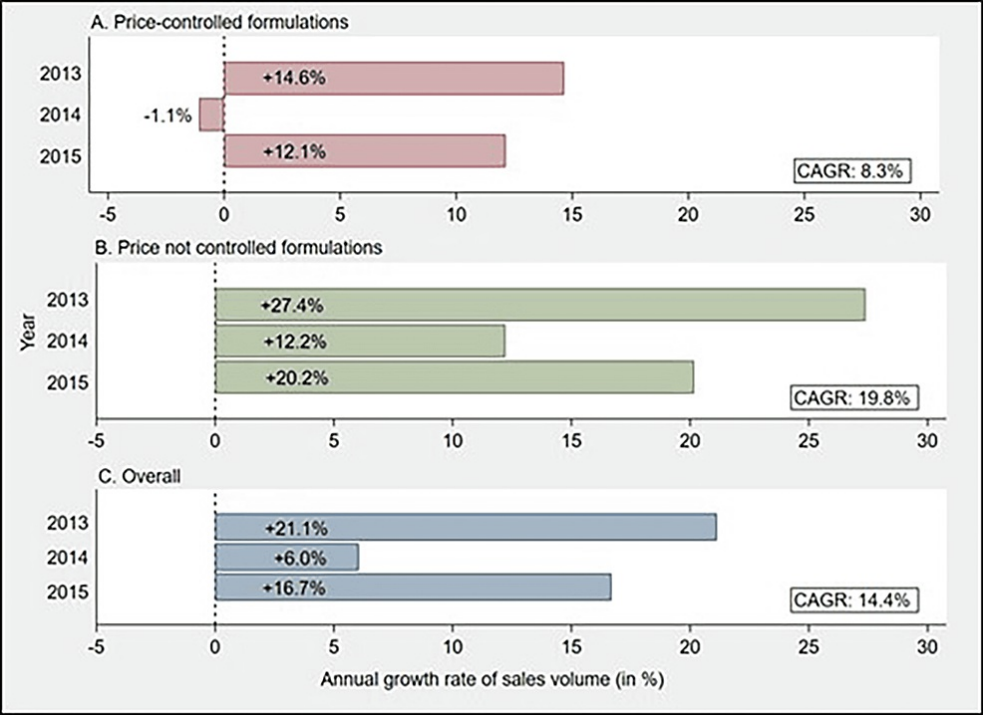
Bar chart showing year-on-year growth rate (in %) and compound annual growth rate (CAGR) (in %) of sales volume for A) price-controlled formulations, B) price not controlled formulations, and C) overall anticancer drugs CAGR- compound annual growth rate

**Figure 2:**
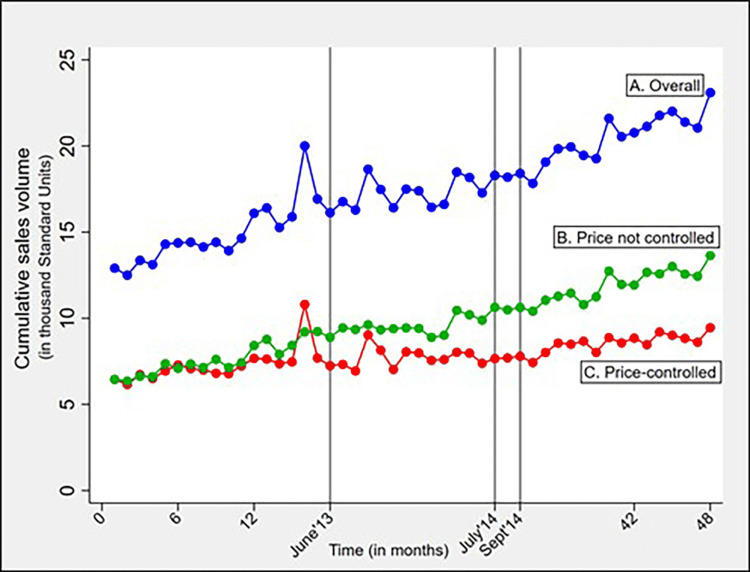
Line chart showing the trend of total monthly sales volume (in thousand Standard Units) for the total study period (Jan-12 to Dec-15) for A) overall anticancer drugs, B) price not controlled formulations, and C) price-controlled formulations Vertical lines (in grey) represent the first ceiling price notification on Jun-13, the last ceiling price notification on Jul-14 and 45-day adjustment period (Aug-14 to Sep-14)

The cumulative utilization of price-controlled formulations in 2015 was 22.4 thousand SUs higher than that in 2012 (a relative increase of 27.2%) (Figure 1). In terms of monthly utilization, the growth of price-controlled formulations became stagnant as DPCO 2013 came into force (Figure 2). While the manufacturers were revising the maximum retail prices of formulations in the adjustment period (August 2014 to September 2014), the price-controlled market showed a negligible reduction or no change in the monthly utilization. But, immediately after the adjustment period, the sales volume of formulations declined minimally.

**Figure 3:**
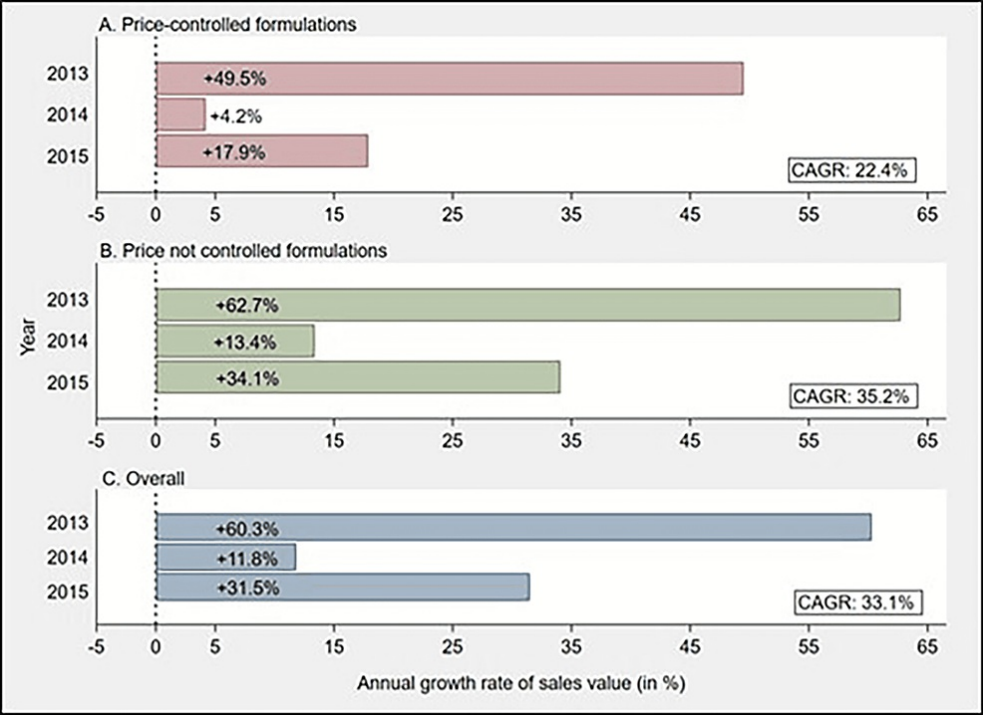
Bar chart showing year-on-year growth rate (in %) and CAGR (in %) of sales value for A) price-controlled formulations, B) price not controlled formulations, and C) overall anticancer drugs CAGR- compound annual growth rate

**Figure 4:**
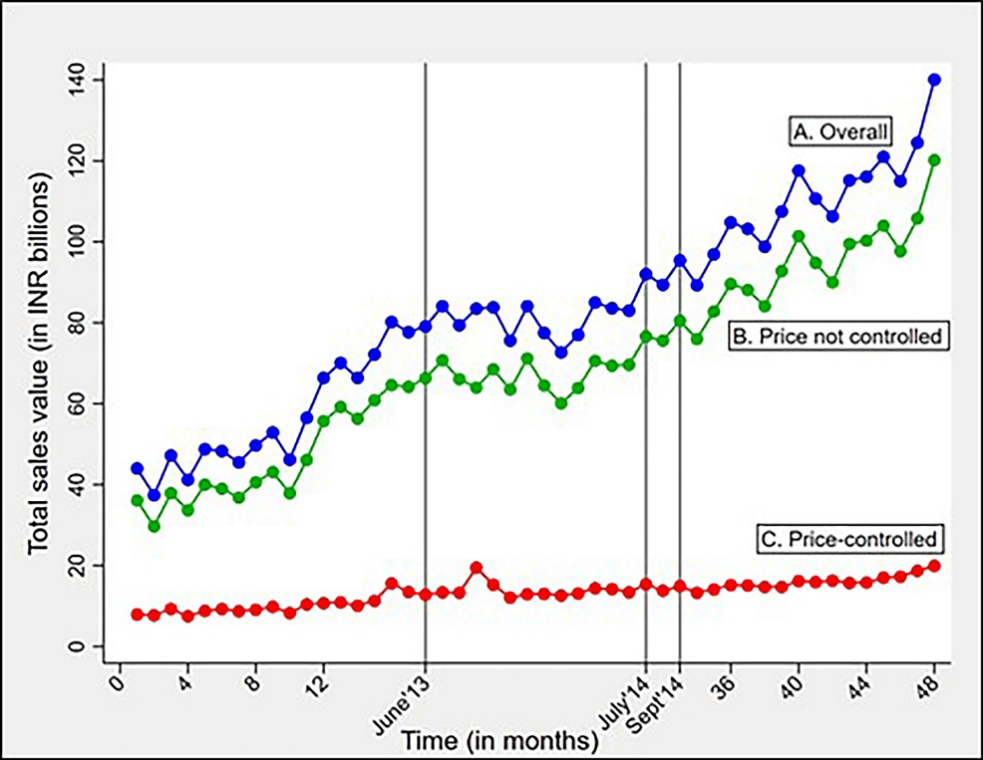
Line chart showing the trend of total monthly sales value (in INR billions) for the total study period (Jan-12 to Dec-15) for A) overall anticancer drugs, B) price not controlled formulations, and C) price-controlled formulations Vertical lines (in grey) represent first ceiling price notification on Jun-13, last ceiling price notification on Jul-14 and 45-day adjustment period (Aug-14 to Sep-14). INR- Indian Rupee

The cumulative sales value of price-controlled formulations increased throughout the study period, as suggested by consistently positive year-on-year growth rates, with the least growth seen in 2014 (Figure 3). The cumulative sales value of price-controlled formulations was 89.8 INR billions higher in 2015 than the sales value in 2012, demonstrating a relative increase of 83.5%. The monthly sales value of price-controlled formulations remained stable throughout the study period, with a slight reduction observed during the price-adjustment period (Figure 4).

Similar annual trends were observed in summary measures of sales volume and sales value (Table 1).

**Table 1:** Summary measures of sales volume and sales value for price-controlled, price not controlled, and overall anticancer medicines ^a^Mean sales volume (SD) and Median sales value (interquartile range) reported. SD- Standard Deviation, INR- Indian Rupee

	Sales volume (in thousand standard units)				Sales value (in INR billions)			
	2012	2013	2014	2015	2012	2013	2014	2015
Price-controlled formulations (n=347), Mean (SD)	6.9 (0.41)	7.9 (1.08)	7.8 (0.33)	8.8 (0.37)	8.9 (1.02)	13.4 (2.51)	13.9 (0.91)	16.4 (1.57)
Price not controlled formulations (n=1209), Mean (SD)	7.1 (0.58)	9.1 (0.50)	10.2 (0.76)	12.3 (0.80)	39.7 (6.56)	64.6 (4.42)	73.3 (8.57)	98.2 (9.54)
Overall anticancer medicines (n=1556)^a^	7.0 (0.51)	8.5 (1.02)	9.0 (1.35)	10.5 (1.89)	20.2 (29.5)	37.9 (51.3)	37.7 (59.15)	52 (82.55)

**Figure 5:**
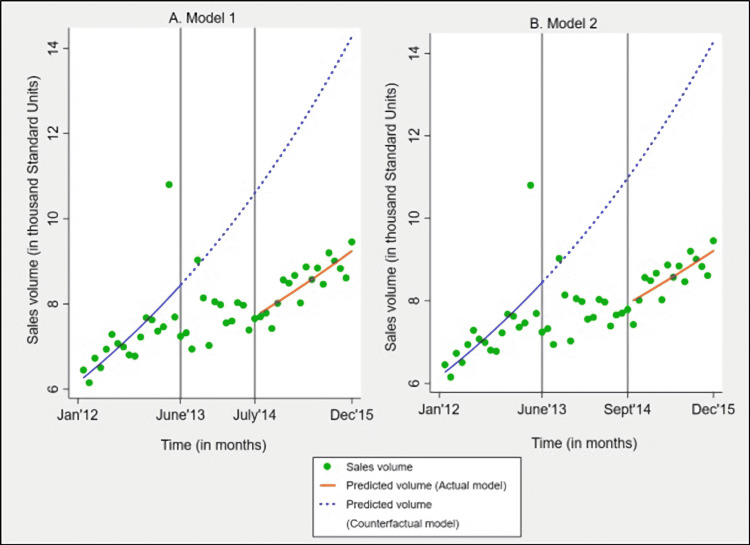
Policy impact on utilization of price-controlled anticancer formulations on A) including the adjustment period utilization (Model 1) and B) excluding adjustment period utilization (Model 2) Absolute policy effect is defined as the difference between the predictions of actual and counterfactual model. Actual model refers to models accounting for intervention and counterfactual model refers to scenario with no intervention. Vertical lines (in grey) represent policy implementation period: Jun-13 to Jul-14 for Model 1 and Jun-13 to Sep-14 for Model 2.

On evaluating the impact of pricing policy on the utilization of price-controlled anticancer formulations (Model 1 results in Table 2 and Figure 5A), we found that at the beginning of the study, the average utilization of price-controlled formulations was 6.2 thousand Standard Units (95% CI 5.7, 6.6). Before the intervention, the utilization was increasing by an average of 1.8% (95% CI 1.1%, 2.5%; p<0.001) every month, independent of the intervention effect, and this trend change was statistically significant. After the intervention was implemented, the utilization of price-controlled formulations immediately reduced by 27.3% (95% CI -38.6%, -13.9%; p=0.001), and this reduction was statistically significant. Results also revealed that post-intervention, the utilization reduced by an average of 0.7% (95% CI -1.6%, 0.3%; p=0.16) every month but this change was statistically non-significant. When comparing the predicted utilization of Model 1 (actual model) against the counterfactual model (based on pre-intervention utilization only), we found that in the final study month, i.e., December 2015, the average utilization of price-controlled anticancer formulations was 5.03 thousand Standard Units lower than what would have been expected without pricing policy (absolute policy effect presented in appendix).

**Table 2:** Regression results showing impact of policy on price-controlled, and price not controlled anticancer formulations ^I^Includes sales volume (utilization) of 45-days price adjustment period. ^E^Excludes sales volume (utilization) of 45-days price adjustment period. ^a^Durbin-Watson test assesses the null hypothesis, H0: no first-order autocorrelation or d≈2 ^b^Breusch-Godfrey test assesses the null hypothesis, H0: no autocorrelation ^c^Absolute policy effect = Predicted sales volume of Actual model minus Predicted sales volume of Counterfactual model. ***P<0.001; **P<0.01; *P<0.05. SUs- Standard Units

Independent variables	Price-controlled		Price not controlled	
	Model 1^I^	Model 2^E^	Model 3^I^	Model 4^E^
Baseline level (β_0_) (in thousand SUs)	6.2*** (5.7, 6.6)	6.2*** (5.7, 6.6)	6.2*** (5.9, 6.4)	6.2*** (5.9, 6.5)
Baseline trend (β_1_)	1.8%*** (1.1, 2.5)	1.8%*** (1.0, 2.5)	2.2%*** (1.8, 2.6)	2.2%*** (1.8, 2.7)
Level change (β_2_)	-27.3%** (-38.6, -13.9)	-27.8%** (-40.3, -12.7)	-16.2%** (-24.1, -7.5)	-17.6%** (-26.3, -7.8)
Trend change (β_3_)	-0.7% (-1.6, 0.3)	-0.7% (-1.8, 0.4)	-0.7%* (-1.3, -0.1)	-0.7%* (-1.3, -0.03)
Number of pre-policy observations	17	17	17	17
Number of post-policy observations	17	15	17	15
R^2^	0.74	0.74	0.98	0.98
Durbin-Watson statistic (d)^a^	2.39	2.37	2.10	2.07
Breusch-Godfrey test p-value^b^	0.18	0.17	0.68	0.71
Absolute policy effect for final study month, i.e., Dec-15 (in thousand SUs)^c^	-5.03	-5.06	-4.58	-4.57

Altering the implementation period for price-controlled formulations (Model 2 results in Table 2 and Figure 5B) resulted in estimates that were comparable to Model 1. The absolute policy effect on the final study month, i.e., December 2015 for Model 2, was -5.06 thousand Standard Units, which was only 0.03 thousand Standard Units higher reduction than that seen in Model 1 (absolute policy effect presented in appendix). We did not detect autocorrelation of any order in our regression models (autocorrelation results of all regression models presented in appendix).

However, the subgroup analysis showed that policy (DPCO 2013) caused a statistically significant change in utilization of seven anticancer molecules, i.e., Alpha interferon, Carboplatin, Cytosine arabinoside, Doxorubicin, Etoposide, Mesna, and Oxaliplatin (Table 3). Among these, six molecules showed an immediate as well as sustained reduction in utilization after the implementation of DPCO 2013. Mesna, on the other hand, showed an immediate decline, followed by a sustained increase in post-policy utilization. Immediately after the implementation of DPCO 2013, Melphalan demonstrated highest reduction in utilization (-83.5%; 95% CI -92.4%, -63.9%; p<0.001), followed by Mesna (-62.2%; 95% CI -79.6%, -29.8%; p=0.003), Chlorambucil (-61.4%l 95% CI -82.1%, 17.0%; p=0.02), and Paclitaxel (-52.3%; 95% CI -74.9%, -9.3%; p=0.03). Although Actinomycin D also showed a substantial immediate reduction in utilization after DPCO 2013 implementation, its level reduction was not significant statistically. In terms of long-term change in utilization, six anticancer molecules showed a statistically significant trend reduction after policy implementation. Alpha interferon showed the maximum trend reduction in post-policy sales volume (-10.3%; 95% CI -14, -6.5; p<0.001), followed by Oxaliplatin (-6.1%; 95% CI -8.7, -3.4; p<0.001), Etoposide (-4.7%; 95% CI -7.2, -2.3; p<0.001), Cytosine arabinoside (-4.7%; 95% CI -7.7, -1.7; p=0.003), Cisplatin (-4.5%; 95% CI -6.2, -2.8; p<0.001), and Daunorubicin (-4%; 95% CI -6.5, -1.4; p=0.003). Of the molecules that showed an increase in utilization trend after policy implementation, Vinblastine sulphate showed the maximum increase in trend (14.4%; 95% CI 8, 21.3; p<0.001), followed by Mesna (8.1%; 95% CI 3.4, 13.1; p=0.001), Vincristine (5.7%; 95% CI 0.4, 11.2; p=0.04), Cyclophosphamide (2.2%; 95% CI 0.2, 4.3; p=0.03), and Flutamide (1.6%; 95% CI 0.3, 2.9; p=0.02).

**Table 3:** Results of subgroup analysis- Segmented regression results for each price-controlled anticancer formulation ^a^Durbin-Watson test assesses the null hypothesis, H0: no first-order autocorrelation or d≈2 ^b^Breusch-Godfrey test assesses the null hypothesis, H0: no autocorrelation ^c^Absolute policy effect = Predicted sales volume of Actual model minus Predicted sales volume of Counterfactual model. ***P<0.001; **P<0.01; *P<0.05. SUs- Standard Units Note: Three anticancer molecules (L-Asparaginase, Dacarbazine, and Busulphan) had inadequate observations, hence their results have not been presented here

Price-controlled anticancer formulation	Baseline level (β_0_) (in SUs)	Baseline trend (β_1_)	Level change (β_2_)	Trend change (β_3_)	Number of observations		R^2^	Durbin-Watson statistic (d)^a^	Breusch-Godfrey test P value^b^	Absolute policy effect for final study month (in SUs)^c^
					Pre-policy	Post-policy				
1) 5-Flurorouracil	23.1*** (18, 29.8)	1.8% (-0.1, 3.7)	-31.6% (-53.5, 0.6)	-1.5% (-4.1, 1.2)	23	22	0.11	1.69	0.63	-27.26
2) Actinomycin D	0.3 (0, 17531.5)	5.5% (-28.5, 55.7)	-63.3% (-96, 234)	2.6% (-31.4, 53.4)	3	9	0.29	1.98	0.35	-1.66
3) Alpha Interferon	1.1 (0.8, 1.6)	8%*** (4.1, 12)	-61.4%** (-76.9, -35.4)	-10.3%*** (-14, -6.5)	16	22	0.59	1.63	0.67	-43.93
4) Azathioprine	2325*** (2187.3, 2471.4)	0.9%** (0.3, 1.5)	-11.1%** (-18.4, -3.1)	0.01% (-0.6, 0.7)	17	28	0.70	2.13	0.43	-393.24
5) Bleomycin	1.2 (0.3, 4.6)	-0.2% (-9.6, 10.1)	-32.7% (-70.1, 51.5)	4.3% (-5.6, 15.4)	7	28	0.38	1.92	0.71	1.31
6) Carboplatin	158.6*** (124.6, 201.8)	6.6%*** (4.1, 9.1)	-38.2%** (-55.9, -13.4)	-3.9%** (-6.4, -1.4)	17	28	0.73	1.83	0.95	-2687.17
7) Chlorambucil	4.0*** (2.4, 6.7)	0.7% (-3.2, 4.7)	-61.4%* (-82.1, -17)	4.7% (-0.7, 10.5)	22	23	0.20	1.77	0.73	0.65
8) Cisplatin	333.2*** (283.7, 391.4)	3.8%*** (2.2, 5.5)	-12.4% (-31, 11.3)	-4.5%*** (-6.2, -2.8)	17	27	0.53	1.51	0.17	-1521.96
9) Cyclophosphamide	208.0*** (174, 248.6)	-0.4% (-2.1, 1.4)	-14.9% (-35.7, 12.7)	2.2%* (0.2, 4.3)	17	26	0.30	1.64	0.26	88.91
10) Cyclosporine	122.1*** (107.9, 138.3)	1.8%** (0.5, 3)	9.7% (-7.9, 30.6)	0.4% (-0.9, 1.8)	17	28	0.89	2.04	0.59	68.50
11) Cytosine arabinoside	23.68*** (17.7, 31.7)	5.9%*** (2.9, 9)	-34.2%* (-56.3, -1)	-4.7%** (-7.7, -1.7)	17	28	0.45	1.54	0.25	-308.1
12) Daunorubicin	2.0*** (1.6, 2.6)	4.1%*** (2.1, 6.1)	-19.7% (-45, 17.3)	-4.0%** (-6.5, -1.4)	22	23	0.43	2.05	0.63	-9.31
13) Doxorubicin	28.2*** (23.4, 34)	3.8%*** (1.9, 5.7)	-37.8%** (-52.1, -19.1)	-2.8%** (-4.7, -0.8)	17	28	0.35	1.60	0.29	-119.51
14) Etoposide	21.5*** (17.2, 26.9)	5.3%*** (3, 7.6)	-40.4%* (-60.5, -10.2)	-4.7%*** (-7.2, -2.3)	17	23	0.57	1.13	0.01	-203.74
15) Flutamide	100.2*** (88.7, 113.1)	-1.5%* (-2.6, -0.3)	5.4% (-11, 25)	1.6%* (0.3, 2.9)	17	28	0.24	1.62	0.43	31.93
16) Gemcitabine hydrochloride	3.6*** (2.9, 4.5)	4.5%*** (2.2, 6.8)	-8.2% (-33.3, 26.4)	-2.0% (-4.4, 0.5)	17	28	0.79	1.50	0.20	-14.18
17) Ifosfamide	1.0 (0.7, 1.4)	0.8% (-1.8, 3.4)	27.6% (-23.7, 113.6)	-2.3% (-5.6, 1.1)	18	22	0.13	2.03	0.54	-0.34
18) Imatinib	50.7*** (36.9, 69.8)	4.1%* (0.9, 7.4)	14.5% (-26.8, 79)	-2.2% (-5.5, 1.2)	17	28	0.68	1.28	0.22	-136.90
19) Melphalan	6.0*** (3.6, 10.1)	3.4% (-0.6, 7.6)	-83.5%*** (-92.4, -63.9)	-1.8% (-7, 3.7)	22	23	0.54	1.75	0.91	-27.17
20) Mercaptopurine	78.2*** (62.1, 98.7)	1.4% (-0.3, 3.3)	-34.3%* (-53.6, -7.1)	1.3% (-1.2, 3.7)	22	23	0.27	1.78	0.52	-19.67
21) Mesna	3.5*** (2.3, 5.3)	0.1% (-3.9, 4.1)	-62.2%** (-79.6, -29.8)	8.1%** (3.4, 13.1)	17	25	0.62	1.75	1.00	7.63
22) Methotrexate	1737.3*** (1538.1, 1962.4)	0.3% (-0.9, 1.5)	-10.4% (-25.3, 7.3)	-0.5% (-1.8, 0.8)	17	27	0.18	0.74	<0.001	-448.81
23) Mitomycin-C	0.9 (0.7, 1.2)	1.4% (-0.5, 3.4)	-31.4% (-53.9, 1.9)	0.7% (-2.1, 3.6)	21	21	0.16	1.49	0.14	-0.36
24) Oxaliplatin	5.0*** (3.8, 6.6)	8.9%*** (6.3, 11.5)	-41.4%** (-60.5, -13.2)	-6.1%*** (-8.7, -3.4)	19	26	0.78	1.80	0.97	-261.86
25) Paclitaxel	2.0** (1.3, 3.1)	4.3%* (1, 7.8)	-52.3%* (-74.9, -9.3)	-1.2% (-5.5, 3.4)	22	23	0.24	1.55	0.23	-9.82
26) Procarbazine	2.4*** (1.6, 3.4)	3.1%* (0.3, 6)	28.7% (-25.3, 121.6)	0.4% (-3.4, 4.2)	22	23	0.69	1.50	0.13	4.11
27) Tamoxifen Citrate	947.9*** (780.9, 1150.7)	3.4%** (1.4, 5.3)	-19.6% (-38.8, 5.5)	-2.9%** (-4.9, -0.9)	17	28	0.37	2.12	0.46	-3009.79
28) Vinblastine sulphate	1.3 (0.7, 2.3)	-0.3% (-4.4, 4.1)	-44.5% (-76, 28.4)	14.4%*** (8, 21.3)	22	23	0.63	0.99	<0.001	12.82
29) Vincristine	1.8** (1.2, 2.8)	-4.9%* (-9.1, -0.4)	66.3% (-22.3, 256.2)	5.7%* (0.4, 11.2)	11	16	0.19	1.18	0.31	1.14

## Discussion

In our study, we used an interrupted-time series design to quantify the impact of the Drug Prices Control Order of 2013 on the utilization of anticancer medicines in the private retail medicine sector of India. We found that utilization showed a slight decline at around a year after DPCO 2013 came into force. A notable finding was a reduction in both sales volume and value during the 45 days price adjustment period, which was consistent with our expectation as during this period, manufacturers would have withdrawn drug packs from the market for re-labeling, causing a reduction in sales. Segmented regression revealed that the baseline trend of utilization of anticancer medicines was increasing before the policy was implemented. But, DPCO 2013 caused a reduction in the utilization of price-controlled anticancer formulations, most of which belonged to the cytotoxic drug class, as suggested by our subgroup analysis results.

Subgroup analysis revealed that immediately after the policy was implemented, melphalan showed the most substantial reduction in utilization, and alpha-interferon showed the highest long-term reduction in utilization in the post-policy period.

There could be two plausible explanations for the reduction in utilization observed in our study. Firstly, certain features of DPCO 2013, such as controlling prices of specific strengths and dosage forms of a medicine and only regulating single-dose formulations, could have been used to selectively prescribe or market certain formulations which were not under price control policy, thus influencing the prescription practice. Secondly, our study data was not an exhaustive source of the utilization of anticancer medicines in India. Our data source lacked sales data for eight essential anticancer medicines. Moreover, it was a stockist level data, which is just one of the many procurement sources of medicines in hospitals, where most cancer treatment is administered. Thus, our results hold true only for the anticancer market that was surveyed by our data source.

Similar to our results, a study conducted by Guan et al. in China in 2019 showed that controlling maximum retail prices of anticancer medicines reduced the post-policy trend of utilization of regulated medicines [12]. Another study conducted in the U.S. by Leiberman et al. in 2015 used an interrupted time series design to assess the impact of prescription capping of overall medicines on utilization of essential medicines and found that the policy reduced the proportion of essential medicine prescription [25].

Our study results on the impact of price control on anticancer medicines were different from the impact of DPCO 2013 on statins, where a shift in utilization from price-not-controlled statins to price-controlled statins was reported [21]. This is due to the difference in nature of illness and the medicines required to treat such conditions, as, unlike anticancer medicines, statins are substitutable, and prescribers can change their prescription behavior for diseases associated with statin use. A study conducted in the U.S. by Dusetzina et al. in 2018 used a difference-in-difference approach to assess the impact of a state parity law, providing full insurance coverage of chemotherapy. Although this study showed an increase in the utilization of anticancer medicines, it was not found to be associated with the policy [18]. A study in China by Diao et al., conducted in 2019, found that insurance coverage of six targeted anticancer medicines improved their utilization [19]. Another Chinese study conducted by Hsu et al. in 2019 used an interrupted time series design and found that modifications of reimbursement policy (coverage of erlotinib and gefitinib) caused an increase in the prescription rate of these targeted therapies [20]. A plausible reason for the disparity of our study results with the results of non-Indian origin studies could be varying prescription practices, health system factors, and strength of governance across countries worldwide. 

A major limitation of our study was that we could not assess the impact of the policy (DPCO 2013) on the utilization of current price-controlled formulations (those listed in NLEM 2015), as our study data had inadequate data points beyond 2015. In addition, our study data lacked patient-level information; thus, we could not assess patient-level prescription use, and we could not adjust for varying patient characteristics during the study period. Moreover, we could not correlate the market findings with any clinically relevant health outcomes.

## Conclusions

The evidence generated in our study suggested that before the policy was implemented, utilization of anticancer medicines had an increasing trend. But the Drug Prices Control Order of 2013 caused an immediate as well as long-term decline in the utilization of anticancer medicines in the private pharmaceutical sector of India. However, our study data was limited to a specific part of the entire anticancer drug market of India, which must be considered when interpreting our findings.

## Figures and Tables

**Figure 6:**
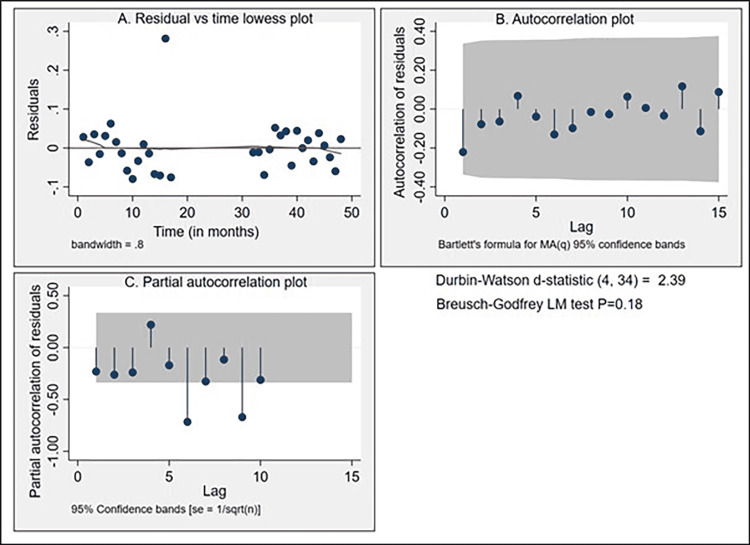
Autocorrelation test results for Model 1 (price-controlled formulations) Model 1 tests policy impact on price-controlled formulations only and includes utilization of 45-day price adjustment period. Intervention implementation period- Jun-13 to Jul-14.

**Figure 7:**
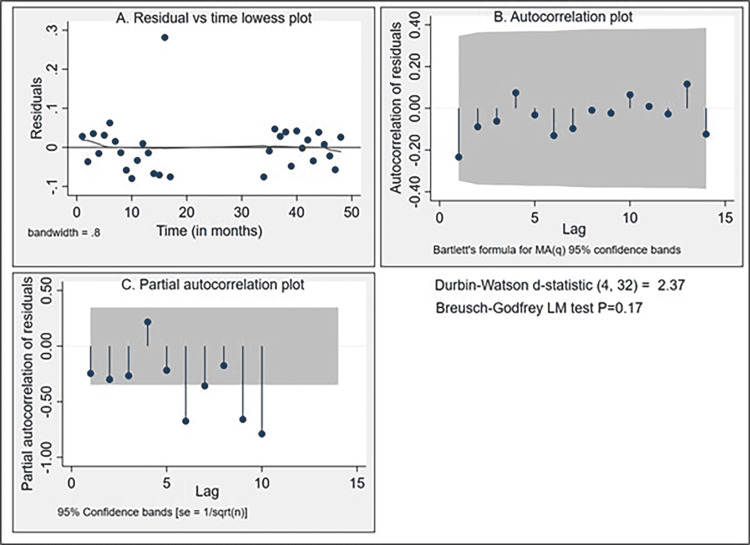
Autocorrelation test results for Model 2 (price-controlled formulations) Model 2 tests policy impact on price-controlled formulations only and excludes utilization of 45-day price adjustment period. Intervention implementation period- Jun-13 to Sep-14.

**Figure 8:**
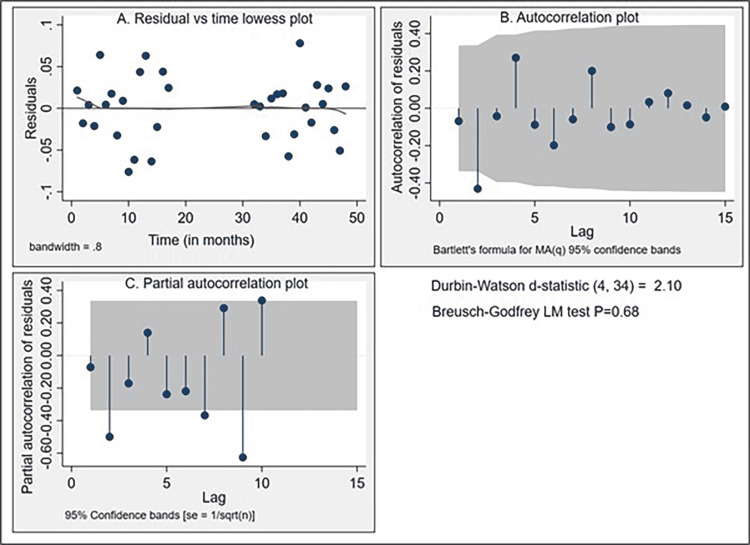
Autocorrelation test results for Model 3 (price not controlled formulations) Model 3 tests policy impact on price not controlled formulations only and includes utilization of 45-day price adjustment period. Implementation period- Jun-13 to Jul-14.

**Figure 9:**
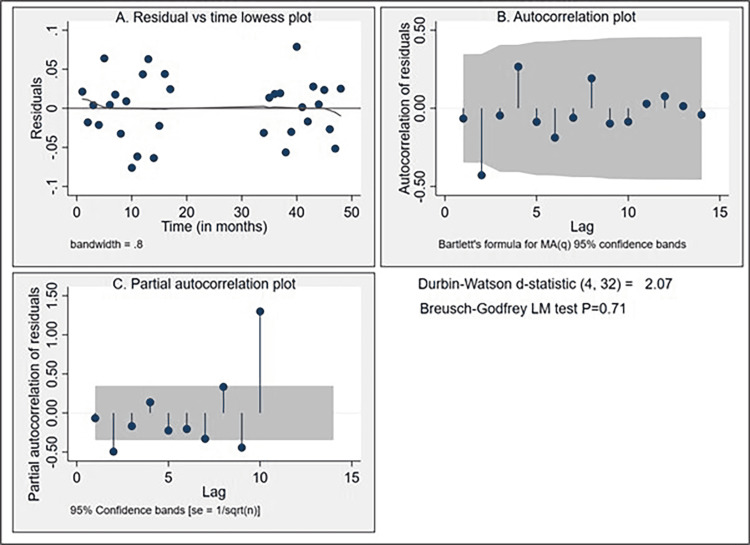
Autocorrelation test results for Model 4 (price not controlled formulations) Model 4 tests policy effect on price not controlled formulations only and excludes utilization of 45-day price adjustment period. Intervention implementation period- Jun-13 to Sep-14.

## Appendices

**Table 4:** List of included and excluded essential anticancer formulations, with the ceiling prices and notification dates Table shows that our study data included 45 essential anticancer formulations, most of them being cytotoxic drugs. Majority of ceiling prices were notified in 2013, earliest being June 2013. Only 1 formulation (Actinomycin D) had its ceiling price notified in July 2014. Thus, ceiling price notification period of pricing policy was June 2013 till July 2014. Our study data did not include 18 essential anticancer formulations. Most of the excluded formulations are indicated for palliative care and management of chemotherapy-induced toxicity.

S. No.	Name of drug	Formulation		Strengths	Notification date	Ceiling price per unit formulation as of 2020	Drug class	Drug sub-class
Essential anticancer formulations included in study data								
1	5-Fluorouracil	Injection		250 mg / 5 ml	2nd December 2013	₹ 2.08	Cytotoxic drug	Antimetabolites (Pyrimidine antagonist)
2	Actinomycin D/ Dactinomycin	Injection		0.5 mg	10th July 2014	₹ 562.98	Cytotoxic drug	Antitumor antibiotics
3	Alpha Interferon	Injection		3 million IU	14th June 2013	₹ 759.70	Toxicity amelioration/ Palliative care	Immunomodulatory (antiviral)
4	Azathioprine	Tablets		50 mg	14th June 2013	₹ 9.89	Cytotoxic drug	Antimetabolites (Purine antagonist)
5	Bleomycin	Injection		15 mg	14th June 2013	₹ 616.06	Cytotoxic drug	Antitumor antibiotics
6	Busulphan	Tablets		2 mg	5th November 2013	₹ 3.41	Cytotoxic drug	Alkylating agents
7	Carboplatin	Injection		150 mg	28th June 2013	₹ 789.01	Cytotoxic drug	Platinum coordination complexes
8	Carboplatin	Injection		450 mg vial	28th June 2013	₹ 2,424.93	Cytotoxic drug	Platinum coordination complexes
9	Chlorambucil	Tablets		2 mg	5th November 2013	₹ 45.22	Cytotoxic drug	Alkylating agents
10	Cisplatin	Injection		10 mg / vial	22nd July 2013	₹ 84.84	Cytotoxic drug	Platinum coordination complexes
11	Cisplatin	Injection		50 mg / vial	28th June 2013	₹ 301.26	Cytotoxic drug	Platinum coordination complexes
12	Cyclophosphamide	Tablets		50 mg	14th June 2013	₹ 3.75	Cytotoxic drug	Alkylating agents
13	Cyclophosphamide	Injection		500 mg	21st August 2013	₹ 72.71	Cytotoxic drug	Alkylating agents
14	Cyclosporine	Capsules		25 mg	14th June 2013	₹ 24.75	Toxicity amelioration/ Palliative care	Immunosuppressant
15	Cyclosporine	Capsules		50 mg	14th June 2013	₹ 48.26	Toxicity amelioration/ Palliative care	Immunosuppressant
16	Cyclosporine	Capsules		100 mg	28th June 2013	₹ 106.29	Toxicity amelioration/ Palliative care	Immunosuppressant
17	Cytosine arabinoside/ Cytarabine	Injection		100 mg/vial	28th June 2013	₹ 238.44	Cytotoxic drug	Antimetabolites (Pyrimidine antagonist)
18	Cytosine arabinoside/ Cytarabine	Injection		500 mg/vial	28th June 2013	₹ 515.53	Cytotoxic drug	Antimetabolites (Pyrimidine antagonist)
19	Cytosine arabinoside/ Cytarabine	Injection		1000 mg/vial	28th June 2013	₹ 1,170.62	Cytotoxic drug	Antimetabolites (Pyrimidine antagonist)
20	Dacarbazine	Injection		500 mg	14th June 2013	₹ 1,029.09	Cytotoxic drug	Alkylating agents
21	Daunorubicin	Injection		20 mg vial	5th November 2013	₹ 353.69	Cytotoxic drug	Antitumor antibiotics
22	Doxorubicin	Injection		10 mg	28th June 2013	₹ 196.48	Cytotoxic drug	Antitumor antibiotics
23	Etoposide	Capsules		100 mg	5th November 2013	₹ 52.30	Cytotoxic drug	Topoisomerase-2 inhibitors
24	Etoposide	Injection		100 mg/ 5 ml vial	28th June 2013	₹ 192.39	Cytotoxic drug	Topoisomerase-2 inhibitors
25	Flutamide	Tablet		250 mg	28th June 2013	₹ 8.62	Hormonal drug	Antiandrogen
26	Gemcitabine hydrochloride	Injection		200 mg	14th June 2013	₹ 1,304.86	Cytotoxic drug	Antimetabolites (Pyrimidine antagonist)
27	Gemcitabine hydrochloride	Injection		1 gm	14th June 2013	₹ 5,969.16	Cytotoxic drug	Antimetabolites (Pyrimidine antagonist)
28	Ifosfamide	Injection		1 gm/2ml vial	2nd December 2013	₹ 344.88	Cytotoxic drug	Alkylating agents
29	Imatinib	Tablets		100 mg	21st June 2013	₹ 87.59	Targeted drug	Tyrosine protein kinase inhibitors
30	Imatinib	Tablets		400 mg	14th June 2013	₹ 268.33	Targeted drug	Tyrosine protein kinase inhibitors
31	L- Asparaginase	Injection		5000 KU.	5th November 2013	₹ 1,183.92	Cytotoxic drug	Miscellaneous
32	Melphalan	Tablet		2 mg	5th November 2013	₹ 108.53	Cytotoxic drug	Alkylating agents
33	Melphalan	Tablet		5 mg	5th November 2013	₹ 182.06	Cytotoxic drug	Alkylating agents
34	Mercaptopurine	Tablet		50 mg	5th November 2013	₹ 8.93	Cytotoxic drug	Antimetabolites (Purine antagonist)
35	Mesna	Injection		200 mg	22nd July 2013	₹ 24.57	Toxicity amelioration/ Palliative care	Uroprotective thiol agent
36	Methotrexate	Tablet		2.5 mg	14th June 2013	₹ 4.72	Cytotoxic drug	Antimetabolites (Folate antagonist)
37	Methotrexate	Injection		50 mg / ml	22nd July 2013	₹ 33.18	Cytotoxic drug	Antimetabolites (Folate antagonist)
38	Mitomycin-C	Injection		10 mg	2nd December 2013	₹ 397.57	Cytotoxic drug	Antitumor antibiotics
39	Oxaliplatin	Injection		50 mg vial	21st August 2013	₹ 2,993.26	Cytotoxic drug	Platinum coordination complexes
40	Paclitaxel	Injection		30 mg / 5 ml	5th November 2013	₹ 301.24	Cytotoxic drug	Microtubule damaging agents (Taxanes)
41	Procarbazine	Capsules		50 mg	5th November 2013	₹ 31.64	Cytotoxic drug	Alkylating agents
42	Tamoxifen Citrate	Tablets		10 mg	28th June 2013	₹ 4.35	Hormonal drug	Selective oestrogen receptor modulators
43	Tamoxifen Citrate	Tablets		20 mg	14th June 2013	₹ 2.77	Hormonal drug	Selective oestrogen receptor modulators
44	Vinblastine sulphate	Injection		10 mg	5th November 2013	₹ 284.90	Cytotoxic drug	Microtubule damaging agents (Vinca Alkaloids)
45	Vincristine	Injection		1 mg / ml	14th June 2013	₹ 50.60	Cytotoxic drug	Microtubule damaging agents (Vinca Alkaloids)
Essential anticancer formulations absent in study data								
1	Allopurinol		Tablets 100 mg					
2	Cyclophosphamide		Tablets 200 mg					
3	Cyclosporine		Capsules 10 mg					
4	Cyclosporine		Concentrate for injection 100 mg/ml					
5	Danazol		Capsules 50 mg					
6	Danazol		Capsules 100 mg					
7	Filgrastim		Injection 1 ml vial					
8	Folinic Acid		Injection 3 mg / ml					
9	Mercaptopurine		Injection 100 mg / ml					
10	Morphine Sulphate		Tablets 10 mg					
11	Ondansetron		Tablets 4 mg					
12	Ondansetron		Tablets 8 mg					
13	Ondansetron		Injection 2 mg/ml					
14	Ondansetron		Syrup 2 mg/5 ml					
15	Prednisolone		Tablets 5 mg					
16	Prednisolone		Injection 20 mg (as sodium phosphate or succinate)					
17	Prednisolone		Injection 25 mg (as sodium phosphate or succinate)					
18	Raloxifene		Tablets 60 mg					

**Table 5:** Absolute policy effect on price-controlled formulations (in thousand Standard Units) Predicted utilization of actual models were based on Model 1 (testing policy effect on price-controlled formulations and includes price adjustment period utilization) and Model 2 (testing policy effect on price-controlled formulations after excluding price adjustment period utilization). Counterfactual model considers a scenario with no intervention and assumes that in such a scenario, the pre-intervention trend would continue unaltered. Hence, counterfactual model utilization was predicted by regressing time on pre-intervention utilization only. Absolute policy effect refers to change in utilization brought by intervention, as compared to the trend that would have continued without intervention. Thus, it was computed by subtracting counterfactual model predictions from actual model predictions. All utilization figures are in thousand Standard Units.

Month	Predicted utilization of counterfactual model (common for Model 1 and 2)	Model 1		Model 2	
		Predicted utilization of actual model	Absolute policy effect (actual predictions minus counterfactual predictions)	Predicted utilization of actual model	Absolute policy effect (actual predictions minus counterfactual predictions)
Jun-13	8.44	Implementation period: Jun-13 till Jul-14	.	Implementation period: Jun-13 till Sep-14	.
Jul-13	8.59		.		.
Aug-13	8.74		.		.
Sep-13	8.90		.		.
Oct-13	9.05		.		.
Nov-13	9.21		.		.
Dec-13	9.38		.		.
Jan-14	9.54		.		.
Feb-14	9.71		.		.
Mar-14	9.88		.		.
Apr-14	10.06		.		.
May-14	10.23		.		.
Jun-14	10.41		.		.
Jul-14	10.60		.		.
Aug-14	10.79	7.79	-3.00		.
Sep-14	10.98	7.87	-3.10		.
Oct-14	11.17	7.96	-3.21	8.01	-3.16
Nov-14	11.37	8.04	-3.32	8.09	-3.28
Dec-14	11.57	8.13	-3.44	8.17	-3.40
Jan-15	11.77	8.22	-3.55	8.25	-3.52
Feb-15	11.98	8.30	-3.67	8.33	-3.65
Mar-15	12.19	8.39	-3.80	8.42	-3.77
Apr-15	12.41	8.48	-3.92	8.50	-3.90
May-15	12.63	8.58	-4.05	8.59	-4.04
Jun-15	12.85	8.67	-4.18	8.67	-4.17
Jul-15	13.07	8.76	-4.31	8.76	-4.31
Aug-15	13.31	8.85	-4.45	8.85	-4.46
Sep-15	13.54	8.95	-4.59	8.94	-4.60
Oct-15	13.78	9.05	-4.73	9.03	-4.75
Nov-15	14.02	9.14	-4.88	9.12	-4.90
Dec-15	14.27	9.24	-5.03	9.21	-5.06

**Table 6:** Absolute policy effect on price not controlled formulations (in thousand Standard Units) Model 3 tests policy effect on price not controlled formulations and includes price adjustment period utilization. Model 4 tests policy effect on price not controlled formulations after excluding price adjustment period utilization. All utilization figures are in thousand Standard Units.

Month	Predicted utilization of counterfactual model (common for Model 3 and 4)	Model 3		Model 4	
		Predicted utilization of actual model	Absolute policy effect (actual predictions minus counterfactual predictions)	Predicted utilization of actual model	Absolute policy effect (actual predictions minus counterfactual predictions)
Jun-13	9.21	Implementation period: Jun-13 till Jul-14	.	Implementation period: Jun-13 till Sep-14	.
Jul-13	9.41		.		.
Aug-13	9.62		.		.
Sep-13	9.84		.		.
Oct-13	10.06		.		.
Nov-13	10.28		.		.
Dec-13	10.51		.		.
Jan-14	10.75		.		.
Feb-14	10.99		.		.
Mar-14	11.23		.		.
Apr-14	11.48		.		.
May-14	11.74		.		.
Jun-14	12.00		.		.
Jul-14	12.27		.		.
Aug-14	12.54	10.44	-2.11		.
Sep-14	12.83	10.60	-2.23		.
Oct-14	13.11	10.76	-2.35	10.74	-2.38
Nov-14	13.41	10.92	-2.48	10.90	-2.50
Dec-14	13.70	11.09	-2.62	11.07	-2.63
Jan-15	14.01	11.26	-2.75	11.24	-2.77
Feb-15	14.32	11.43	-2.90	11.41	-2.91
Mar-15	14.64	11.60	-3.04	11.59	-3.05
Apr-15	14.97	11.78	-3.19	11.77	-3.20
May-15	15.31	11.96	-3.35	11.95	-3.36
Jun-15	15.65	12.14	-3.51	12.13	-3.51
Jul-15	16.00	12.32	-3.68	12.32	-3.68
Aug-15	16.36	12.51	-3.85	12.51	-3.84
Sep-15	16.72	12.70	-4.02	12.70	-4.02
Oct-15	17.10	12.89	-4.20	12.90	-4.20
Nov-15	17.48	13.09	-4.39	13.10	-4.38
Dec-15	17.87	13.29	-4.58	13.30	-4.57
